# Novel Curcumin-Encapsulated *α*-Tocopherol Nanoemulsion System and Its Potential Application for Wound Healing in Diabetic Animals

**DOI:** 10.1155/2022/7669255

**Published:** 2022-09-15

**Authors:** Muhammad Ali, Nauman Rahim Khan, Zakia Subhan, Saima Mehmood, Adnan Amin, Imran Rabbani, Fazal-ur -Rehman, Hafiz Muhammad Basit, Haroon Khalid Syed, Ikram Ullah Khan, Muhammad Shuaib Khan, Sehrish Khattak

**Affiliations:** ^1^Gomal Centre for Skin/Regenerative Medicine and Drug Delivery Research (GCSRDDR), Faculty of Pharmacy, Gomal University, Dera Ismail Khan, 29050 KPK, Pakistan; ^2^Department of Pharmacy, Kohat University of Science and Technology, Kohat, 26000 KPK, Pakistan; ^3^Kohat Institute of Medical Sciences, Khyber Medical University, Kohat, 26000 KPK, Pakistan; ^4^Department of Pharmacognosy/Natural Product Research Lab, Faculty of Pharmacy, Gomal University, Dera Ismail Khan, 29050 KPK, Pakistan; ^5^Department of Pharmaceutical Chemistry, Faculty of Pharmacy, Gomal University, Dera Ismail Khan, 29050 KPK, Pakistan; ^6^Akhtar Saeed College of Pharmacy, Bahria Golf City, Rawalpindi, Punjab, Pakistan; ^7^Department of Pharmaceutics, Faculty of Pharmaceutical Sciences, Government College University, 38000 Faisalabad, Pakistan; ^8^Department of Basic Veterinary Sciences, Gomal University, Dera Ismail Khan, 29050 KPK, Pakistan; ^9^Department of Microbiology, Kohat University of Science and Technology, Kohat, 26000 KPK, Pakistan

## Abstract

**Objective:**

This project was aimed at formulating a novel nanoemulsion system and evaluating it for open incision wound healing in diabetic animals.

**Methods:**

The nanoemulsions were characterized for droplet size and surface charge, drug content, antioxidant and antimicrobial profiling, and wound healing potential in diabetic animals. The skin samples excised were also analyzed for histology, mechanical strength, and vibrational and thermal analysis.

**Results:**

The optimized nanoemulsion (CR-NE-II) exhibited droplet size of26.76 ± 0.9 nm with negative surface charge (−10.86 ± 1.06 mV), was homogenously dispersed with drug content of68.05 ± 1.2%, released almost82.95 ± 2.2%of the drug within first 2 h of experiment with synergistic antioxidant (95 ± 2.1%) and synergistic antimicrobial activity against selected bacterial strains in comparison to blank nanoemulsion, and promoted significantly fast percent reepithelization (96.47%). The histological, vibrational, thermal, and strength analysis of selected skin samples depicted a uniform and even distribution of collagen fibers which translated into significant increase in strength of skin samples in comparison to the control group.

**Conclusions:**

The optimized nanoemulsion system significantly downregulated the oxidative stress, enhanced collagen deposition, and precluded bacterial contamination of wound, thus accelerating the skin tissue regeneration process.

## 1. Introduction

Diabetes is a rapidly progressing health concern associated with adjuvant manifestations including nephropathy, neuropathy, and retinopathy and is also associated with delayed wound healing, often characterized as a chronic wound with a frustrated and extended healing process [1], where almost 80% of nonhealing diabetic foot ulcers resolve with amputation of the affected body area due to unavailability of satisfactory treatment strategies [2]. Additionally, 80% of diabetic wounds are prone to more complications due to secondary bacterial infections, which results in significant loss of mobility, functions, and quality of patient's life [3]. The annual cost of diabetic wound treatment is estimated to be 20 billion dollars, which is increasing gradually at an annual growth rate of 4% [4]. Wound healing is a complex and intertwined process consisting of four overlapping phases, including homeostasis, inflammation, proliferation, and remodeling [5]. All four phases further consist of various processes that occur in a systematic and adequate manner [6]. Patients with diabetes typically have impaired wound healing due to vascular dysfunction, high oxidative stress, nerve hypoxia, impaired angiogenesis, defective collagen deposition, and weak immune response [7]. When the integumentary system is compromised, one or more phase of wound healing is likewise impaired. Nonhealing wounds are stagnant in one of these stages, typically the inflammation and proliferation stages [8].

Free radical formation in diabetes by nonenzymatic glycation of proteins, glucose oxidation, and increased lipid peroxidation leads to damage of endogenous antioxidant enzymes and cellular machinery and also increased insulin resistance due to oxidative stress [9]. During the mitochondrial oxidation process, high blood glucose leads to excess glycolysis inside the cells, producing much energy in the mitochondria in the form of ATP, and as a by-product, reactive oxygen species (ROS) are produced. [10], and hence, prolonged exposure to free radicals (ROS) is believed to lead to cellular injury and apoptosis via the destructive oxidation of intracellular proteins, lipids, and nucleic acids [11]. Furthermore, hyperglycemia impairs the normal wound healing process due to prolonged inflammatory phase, and hence persistently, high blood glucose levels attract opportunistic bacteria causing localized bacterial infection and could even lead to septicemia, which further compromises the progress of the inflammatory phase [12].

To date, diabetic wound healing strategies involve the use of oral hypoglycemics and/or injectable insulin to keep blood glycemic conditions in optimal condition and topical and/or oral antimicrobials to prevent bacterial infiltration [13], which not only may lead to an extra financial burden on patients, especially in economically compromised countries, but may also result in reducing patient compliance to multiple drug therapy [14].

Curcumin is the miraculous molecule found in turmeric, which has long been used in spices in Asian countries. Curcumin is reported to possess good antioxidant [15], antimicrobial [16], anti-inflammatory [17], anticancerous [18], antidiabetic [19], anticoagulant [20], antifungal [21], antimutagenic [22], antiviral [23], and wound healing properties [24]. Curcumin has also been widely investigated to promote the skin tissue regeneration process and formulated in the form of gels [25], nanoparticles [26], emulsions [27], nanofibers [28], hydrogel [29], and liposomes [30], where it has been reported to significantly enhance tissue remodeling, tissue granulation, and collagen deposition [31]. Vitamin E is a fat-soluble vitamin and is a mixture of *α*-tocopherol and *γ*-tocotrienol, where the former being the most abundant form and has been utilized for its antioxidant activity [32] antibacterial activity [33], anti-inflammatory activity [34], and scar management [35] properties. Vitamin E is a potent chain-breaking antioxidant and helps protect the cells from the oxidative damage elicited by free radicals produced inside the cells in response to the metabolic process taking place inside the cell [36].

The delayed wound healing in people with diabetes makes it imperative to develop a treatment strategy with the inherent ability to reduce the oxidative stress by scavenging the free radicals but also inhibit the microbial growth by exerting antimicrobial activity, with efficient local hypoglycemic effect having the ability to improve the neovascularization (angiogenesis) at the wound site. This study proposes to develop a novel curcumin and vitamin E nanoemulsion system to improve the aqueous solubility, stability, and therapeutic efficacy of curcumin, having synergistic antioxidant, antimicrobial, and hypoglycemic effect and efficient wound healing ability in diabetic animals.

## 2. Materials and Methods

### 2.1. Materials

Curcumin 95% purity was purchased from Ceres Biotech Ltd., Zhejiang, China; tween-80 was graciously supplied by BioLabs Islamabad Pakistan. Polyethylene glycol (PEG-400), *α*-tocopherol acetate, ethanol, methanol, and acetonitrile (Sigma-Aldrich, USA) were purchased. The selected bacterial strains for antimicrobial assay were graciously provided by the Natural Product Research Laboratory, Faculty of Pharmacy, Gomal University, Pakistan (*Staphylococcus aureus* (ATCC # 33862) and *Pseudomonas aeruginosa* (ATCC # 15442)). All chemicals were used without any further purification.

### 2.2. Preparation of Curcumin Nanoemulsions (CR-NE)

The CR-NE was prepared using a high-speed homogenization technique. Briefly, appropriate amount of curcumin was predissolved in PEG-400 under continuous magnetic stirring followed by the addition of tween-80 and thoroughly mixed/dissolved. The mixture was then preceded by the addition of *α*-tocopherol in small increments till complete mixing. The water phase preheated at 45 ± 2°C was added slowly dropwise to the oil phase under continuous magnetic stirring for one hour, followed by homogenization using a high-speed homogenizer (ULTRA-TURRAX, D7813, Germany) at 10,000 rpm for 2 min. The quantities of different ingredients are varied for optimization [37]. The formulations are given in Table 1.

### 2.3. Droplet Size, Surface Charge, and Polydispersity Index

The particle size, particle size distribution, and surface charge of the samples were determined using dynamic light scattering utilizing the Zetasizer Nano ZS 90 (Malvern Instruments; Worcestershire, UK), equipped with software (version 6.34) and a He-Ne laser preset at a wavelength of 635 nm and static scattering angle of 90 degrees. Briefly, 10 *μ*l of the sample was mixed with 1 ml of deionized water and vortexed for 2 minutes, followed by analysis with a Zetasizer. Each result displayed was measured in triplicate, and the results were averaged [38, 39].

### 2.4. Drug Content

The drug content of nanoemulsions was estimated by adopting the already reported method [40], where 1 ml of nanoemulsion was diluted with 9 ml of absolute ethanol followed by centrifugation (Scilogex, USA) of the mixture at 5000 rpm for 1 min to ensure extraction of curcumin. The samples were filtered through a 0.45 *μ*m nylon membrane syringe filter (Merck Millipore, USA) and analyzed on UHPLC. The curcumin content in the nanoemulsion was measured using high-performance liquid chromatography (Perkin Elmer UHPLC, Shelton, CT, USA) with a column C-18 (Supelco, USA). The analyses were carried out under isocratic conditions, using a mobile phase consisting of acetonitrile and 0.2% acetic acid (80 : 20 *v*/*v*) at a flow rate of 1.0 ml/min. Ten microliters of the sample was injected into the system, and curcumin content was determined using a Flexar FX UV/VIS Detector at a wavelength of 425 nm. The limit of detection, quantification, percent precision, and percent accuracy were calculated accordingly.

### 2.5. Antioxidant Assay

Free radical scavenging activity of optimized nanoemulsion system with and without curcumin was evaluated using 2,2-diphenyl-1-picrylhydrazyl (DPPH) assay using quercetin served as a standard. Compounds with antioxidant activity can reduce the DPPH by donating hydrogen and changing the color from deep violet to light yellow. In this assay, a freshly prepared solution of DPPH (50 *μ*l; 0.1 mM) was added to 50 *μ*l of the test (varying concentrations), followed by incubation in a dark cabinet. After 30 minutes, the absorbance of the sample was measured at 517 nm. The percent inhibition was calculated as

(1) $$\begin{array}{c} \text{Percent inhibition }\left(\%\right)=\frac{1-\text{sample absorbance}}{\text{standard absorbance}}\times 100. \end{array}$$

For all tested concentrations, graphs were plotted between percent inhibition versus concentration used [41].

### 2.6. Antimicrobial Assay

The antibacterial activity of the optimized nanoemulsion system with and without curcumin was determined against pathogenic bacteria using minimum inhibitory concentration (MIC) according to the microdilution method described by Shao et al. [42] with slight modifications. First, an aliquot (50 *μ*l) of two-fold serially diluted nanoemulsion was prepared with distilled water in a 96-microwell plate. Then, the bacteria were inoculated with nutrient broth (50 *μ*l) in all wells to get the final concentration from 1.5 mg/ml to 0.02 mg/ml and then sealed to avoid the evaporation loss. After 24 h incubation at 37°C, resazurin sodium salt (40 *μ*l) was added to all wells and subsequently incubated at 37°C for two hours. The color change was visually observed to evaluate the bacterial growth. MIC was the lowest concentration of curcumin-loaded nanoemulsion, having a full inhibitory effect on the bacterial growth.

### 2.7. In Vitro Drug Release

Franz diffusion cell (Perme Gear, Inc. Model no: 4G-01-00-15-12, diffusion area = 53 cm^2^) was used for the drug release experiment. The receiving compartment of the Franz diffusion cell was filled with 6.4 ml bubble-free phosphate-buffered saline pH 7.4 to mimic wound bed conditions [43] and maintained at 37 ± 0.5°C by continuous flow of thermostatic water through its jackets which was continuously magnetically stirred at 400 rpm. A Tuffryn® membrane (0.2 *μ*m pore size, Sartorius Germany) was affixed as a barrier between Franz diffusion cell's donor and receiving compartment. A total of 1 ml of the optimized nanoemulsion was loaded into the donor compartment. Sample aliquots of 1 ml were withdrawn at regular time intervals of 0, 0.5, 1, 2, 4, 8, 12, 16, 18, 20, and 24 h, where fresh buffer in a volume equal to volume withdrawn was replaced at each sampling interval to maintain sink conditions. The samples withdrawn were filtered through a 0.45 *μ*m membrane filter before analysis on HPLC for drug released estimation. The experiment was conducted in triplicate, and the results were averaged.

Where applicable, the mechanism of drug release was investigated by fitting the drug release data into Weibull function as expressed by

(2) $$\begin{array}{c} F=1-\exp\;\left(-\text{a}{\text{t}}^{b}\right), \end{array}$$

where *F* is the drug fraction released at time *t* and *a* and *b* are constants. *b*, as a shape parameter, is characterized as exponential (*b* = 1), sigmoidal (*b* > 1), or parabolic (*b* < 1).

## 3. Animal Study

### 3.1. Diabetes Induction, Wound Infliction, and Wound Contraction Analysis

Healthy male, Sprague-Dawley rats, aged three months and weighing 200 to 250 g, are acclimatized for seven days in individual housing under 12 h light/dark cycle with deionized water and standard pelletized food given *ad libitum* under ambient conditions. Before diabetes induction, all animals were kept on fasting for 24 h with free access to water, and their fasting blood glucose level was measured using a glucometer (Code-free, SD Biosensor, Korea) followed by diabetes induction by chemical method; viz., a single intraperitoneal injection of streptozotocin (50 mg/kg) freshly prepared in normal saline was used for the purpose [44–46]. All animals were then allowed to be fed on 5% glucose solution filled into water bottles, and their blood glucose levels were monitored for 72 h. Those animals having fasting blood glucose levels ≥10 mmol/l were declared diabetic [44]. All diabetic animals were then randomly divided into two groups with *n* = 8 per group. One group was assigned to the control group and another to the experimental group. All animals were then anaesthetized by a single intramuscular injection of ketamine (100 mg/kg) and xylazine mixture (10 mg/kg), and their dorsal region hairs were carefully shaved using sharp blades. The area for wound infliction was marked, and full-thickness open incision wound was inflicted using surgical scissors. The control group only received standard gauze application, while the experimental group received optimized nanoemulsion application on a daily basis till complete wound closure. All animal experimentation was conducted following the institutional ethics regulations adapting the international guidelines (OECD Environment, Health and Safety). The study protocol was approved by the institutional Ethical Review Board vide reference no. 502/QEC/GU, dated 29/03/2019, Gomal University Pakistan.

The surface morphology of the wound was regularly monitored and recorded. The wounds were photographed with a digital camera (Samsung ST65-China) on days 0, 3, 7, 10, 14, and 21 from a fixed distance to assess the progress of wound closure, while the animals were under anesthesia. The photographs were analyzed by using ImageJ software. Percentage reepithelization that represents the percentage of wound reduction from the wound size at day 0 was calculated using the following relation.

(3) $$\begin{array}{c} \text{Percent reepithelization}=\frac{\left(\text{wound size at time }0\right)-\left(\text{wound size at time }t\right)}{\text{wound size at time }0}\times 100. \end{array}$$

### 3.2. In Vivo Microbial Contamination Test

For spontaneous microbial assay, Muhammad's procedure was used with slight modification [45]. Briefly, the nutrient agar plates were prepared by dissolving 2.5 g of nutrient agar in 100 ml of distilled water and autoclaved. Then, at days 3, 7, 14, and 21, the microbial growth test was performed by swabbing the wound with a sterile swab and inoculating it on the Petri dishes containing presterilized media and incubated overnight at 37°C in an incubator for 24 h. Following incubation, all Petri dishes were visually inspected for the propensity of microbial colonies formation and compared for the purpose of microbial growth inhibition by the optimized formulation.

### 3.3. Skin Histology

Animals were sacrificed by cervical dislocation, and the newly regenerated skin tissue containing the wound was excised on day 14 and subjected to histological studies. Briefly, the skin tissues were fixed in 10% formalin aqueous solution for three days at room temperature. After that, the skin tissues were trimmed, washed, and dehydrated in ethanol, followed by cleansing using xylene and finally embedded in paraffin wax. About 5 *μ*m thick sections of the skins were sectioned by microtome (HM-340E, Microm Inc. USA) and stained with hematoxylin and eosin (H&E stain) as well as Masson's trichrome stain [7, 46]. The slides were then photographed using an inverted microscope fitted with a camera (Optika Microscope B-383FL, Italy), and photographs were analyzed for inflammation and collagen deposition.

### 3.4. ATR-FTIR

The dermis layer of the skin extracted from the control group and curcumin nanoemulsion- (CR-NE-II-) treated group were characterized at a resolution of 16 cm^−1^ over a wavenumber region of 400 to 4000 cm^−1^ with an acquisition time of 2 min using an attenuated total reflectance-Fourier transform infrared spectroscopy (ATR-FTIR) (UATR TWO, Perkin Elmer, UK). The FTIR spectra of the dermis layer of different animal group skin samples were compared with each other to evaluate the extent of collagen deposition. The dermal amide-I (1640 to 1635 cm^−1^) and amide-II (1557 to 1554 cm^−1^) absorbance bands usually originate from the proteins of the dermal layer, and hence, the absorbance ratio of treated and untreated animal groups was used as a novel tool to estimate the collagen and/or protein deposition in the skin samples. At least triplicates were carried out for each sample, and the results were averaged.

### 3.5. Tensiometry

The excised skin samples were also subjected to tensile strength analysis using a texture analyzer (Testometric M-500, UK). Briefly, skin samples in specific dimensions (35 mm length and 53 mm width) were excised from animals of each group and fixed between the upper and lower jaw of the tensiometer. The test and withdrawal speeds were fixed at 5 and 10 mm/s, respectively. The sample were uniaxially pulled with a 30 kg load. Each sample's maximum stress force, stress peak, stress yield, and young modulus were recorded [47]. Each sample was analyzed in triplicate, and the results were averaged.

### 3.6. Thermal Analysis

Thermal analysis of skin samples was performed on differential scanning calorimetry (DSC) (Perkin Elmer, USA) to estimate changes in transition temperature and enthalpy as a function of different treatments applied. For this purpose, an accurately weighed 3 mg of the skin sample was crimped in a standard aluminium pan and subjected to thermal analysis in the temperature range of 30 to 180°C at a heating rate of 10°C/min under constant purging of nitrogen at 40 ml/min. In addition, the transition temperature (Δ*T*) and enthalpy (Δ*H*) of each sample were recorded. Each sample was run three times, and the results were averaged.

## 4. Statistical Analysis

All values are expressed as a mean of three experiments with respective standard deviations. Student's *t*-test and analysis of variance (one-way ANOVA) followed by post hoc analysis by Tukey HSD were used with a level of significance set at *p* < 0.05.

## 5. Results and Discussion

### 5.1. Droplet Size, Surface Charge, and Polydispersity Analysis

**Figure 1:**
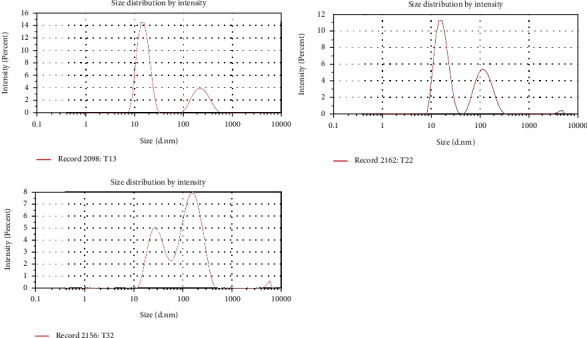
Droplet size and polydispersity index of CR-NE-1, CR-NE-II, and CR-NE-III.

**Figure 2:**
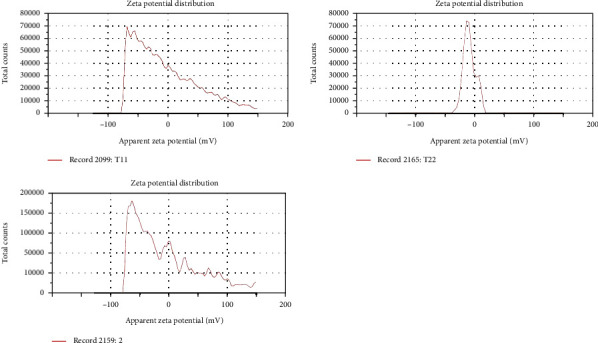
Zeta potential of CR-NE-I, CR-NE-II, and CR-NE-III.

The droplet's size in nanoemulsion directly depends upon the speed of homogenization [48] and the concentration of surfactant [49]. CR-NE had droplet size less than 100 nm due to high shear force applied during homogenization and optimum concentration of surfactant and cosurfactant, which is translated in effectively reducing the interfacial tension between oil and water and also forms a film at the interface between the two immiscible phases to prevent them from recoalescence [50]. In our case, all the formulations showed their droplet size in nanorange Figure 1, which were found to be in the range of 20 to 65 nm, as shown in Table 2. The Pearson correlation analysis depicted that concentration of surfactant (tween-80) is negatively correlated to the size of the droplet (*R* = −0.98, *p* = 0.03) while positively correlated to the surface charge (*R* = 0.11, *p* = 0.45). At the same time, cosurfactant has opposite effect which is positively correlated to the droplet size (*R* = 0.765, *p* = 0.01) but negatively correlated to the surface charge (*R* = −0.97, *p* = 0.15). All the formulations showed a PDI value of less than 1, reflecting uniform size distribution in the formulations [51]. The surface charge of all the formulations was in the range of -0.034 mV to -10 mV, with the latter found for the optimized formulation (CR-NE-II), as shown in Figure 2. The same results is reported by Ahmad et al., who designed a curcumin-loaded nanoemulsion using clove oil as a lipid phase to evaluate its wound repairing and anti-inflammatory activity. The optimized formulation (Cur-NE) exhibited particle diameter 93.64 ± 6.48 nm and PDI = 0.263 ± 0.021, and surface charge was −11.67 ± 0.11 mV. Nanoemulsions are having a small particle size rendering increased surface area which further leads to enhance the release, transfer, and absorption of drugs. Thus, globular size and size distribution and zeta potential are considered as essential parameters for the formulation stability and efficacy [52]. The Pearson correlation analysis revealed that the surface charge is positively related (*R* = 0.99) to concentration of surfactants and cosurfactants and indirect but weak relationship (*R* = 0.76) to concentration of oil phase in the nanoemulsion system. The results are depicted in Table 2.

### 5.2. Drug Content

The drug content of all formulations is given in Table 3. The estimated drug content of all formulations was in the range of 62 and 68%, respectively, dependent on the concentration of surfactant and cosurfactant added. The high variation in the drug content could be attributed to the photodegradation of the drug during the preparatory stage. Curcumin is a highly photosensitive drug, and its efficient encapsulation reduces its chances of degradation [53]. The optimized formulation, i.e., CR-NE-II, contained the surfactant and cosurfactant in the concentration of 10 g and 14 g, respectively. This combination might have formed a droplet enclosing the maximal drug inside the emulsion droplet with additional protection offered by the surfactant layer, which not only help encapsulate the maximal drug inside the core by maximally solubilizing the drug in the oil phase but also protect the drug from photolytic and chemical degradation [54]. The reason for low drug content in other formulations was unknown and not investigated. The limit of detection, limit of quantification, percent precision, and percent accuracy were calculated and found to be 0.28 *μ*g/ml, 0.45 *μ*g/ml, 1.52%, and 98%, respectively.

### 5.3. Antioxidant Activity

**Figure 3:**
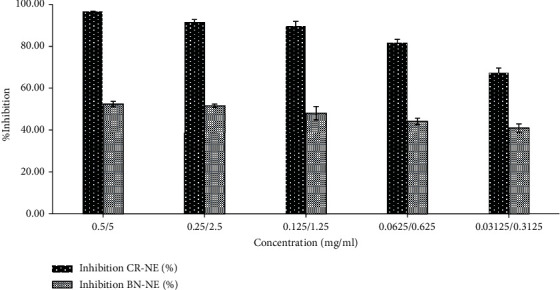
Antioxidant activity of nanoemulsion with and without incorporating curcumin.

The antioxidant activity of curcumin-loaded and blank nanoemulsion was investigated through a DPPH assay. The blank nanoemulsion, despite containing *α*-tocopherol, showed a weak antioxidant profile compared to curcumin-loaded nanoemulsion, where a synergistic antioxidant activity was observed. The percentage inhibition for blank nanoemulsion was found to be 50 ± 4.5% in comparison to CR-NE-II nanoemulsion, where the percentage inhibition was found to be 95 ± 2.1% which was found to be significantly high (Student's *t*-test, *p* < 0.05, Figure 3) at a concentration of 0.5 mg/ml where a dose-dependent antioxidant activity was observed. The antioxidant activity of curcumin is attributed to its unique conjugated structure, which shows typical radical-trapping ability as a chain-breaking antioxidant [55]. Numerous studies reported enhanced antioxidant properties of the curcumin encapsulated in colloidal system such as liposomes [56], polymeric microparticles [57], hydrogel beads [58], nanoparticles [59], and microemulsions [60]. Curcumin containing microemulsion and microemulsion gel was developed with grape seed oil as a lipid phase, where the synergistic antioxidant activity of the optimized microemulsion and gel microemulsions was observed due to the presence of grape seed oil which helped effectively encapsulate and preserve the antioxidant stability of curcumin [61].

The *α*-tocopherol is also a potent lipophilic antioxidant and prevents lipid peroxidation by protonation of fatty acid radicals and hence forms stable phenolic free radicals through its chromane ring [57]. Diabetic wounds are flooded by the high production of reactive oxygen species (ROS) [58], which not only prolong the average healing time but also contribute to local tissue necrosis [59]. The synergistic antioxidant of curcumin and *α*-tocopherol is thus envisaged to exert a local antioxidant effect upon application to diabetic wounds hence contributing to the rapid skin tissue regeneration process.

### 5.4. Antibacterial Activity

The antibacterial activity of curcumin-loaded and blank nanoemulsion was assessed against selected bacterial strains, i.e., *Staphylococcus aureus* (ATCC # 33862) and *Pseudomonas aeruginosa* (ATCC # 15442). The minimum inhibitory concentrations (MIC) of CR-NE-II nanoemulsion and blank nanoemulsion are depicted in Table 4. The MIC for CR-NE-II was 93 *μ*g/ml against *Staphylococcus aureus* while 187.5 *μ*g/ml against *Pseudomonas aeruginosa*, while the blank nanoemulsion did not exert any antibacterial activity at all. Curcumin is a robust antimicrobial agent that interferes with the bacteria's cell membrane and exerts a bactericidal activity [60]. The curcumin encapsulated in the oil phase droplet exerted antibacterial activity by its potential to effectively penetrate the bacterial cell wall, resulting in lysis of peptidoglycan layer, translating into a distortion of the bacterial cell and bacterial cell lysis [61].

In a study, curcumin and quercetin were combined in a 3 different ratio in order to test their antimicrobial, antioxidant, and wound healing activities. The results showed the synergism of the quercetin and curcuminoid combination to inhibit the growth of *S. aureus* and *P. aeruginosa*. The selected mixture containing the ratio 3 : 1 was the optimized formulation in terms of wound healing activity because of its inherent antibacterial, antioxidant, and cell migration-enhancing activities [62]. The exertion of local antimicrobial activity at the wound site by the topical antimicrobial application is considered detrimental to promoting skin tissue regeneration and preventing secondary bacterial infection [63]. Thus, curcumin-loaded *α*-tocopherol nanoemulsion is envisaged to exert local antibacterial action and prevent opportunistic bacteria from infiltrating the wound.

### 5.5. In Vitro Drug Release

**Figure 4:**
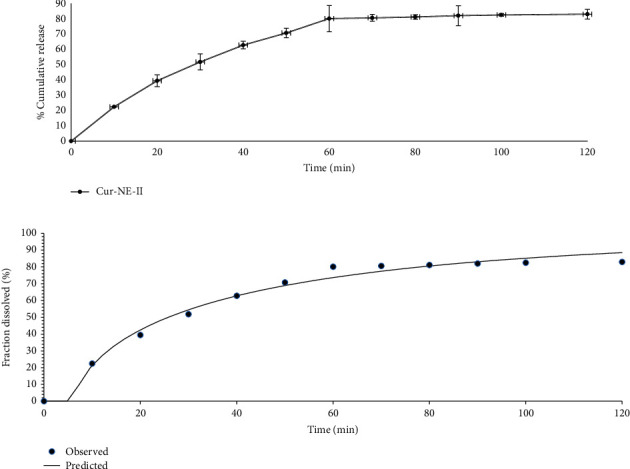
(a) *In vitro* release of curcumin. (b) Weibull kinetics model for drug release mechanism.

The *in vitro* drug release from CR-NE-II nanoemulsion using Tuffryn® membrane as a barrier is shown in Figure 4(a). The nanoemulsion released 82.95% of the drug in the first two hours of the experiment and was then maintained till the end of the experiment. Curcumin is a hydrophobic drug, and its solubility in an aqueous medium can be enhanced by formulating it into a nanoemulsion system [62]. The hydrophilicity of curcumin can further be increased if it is formulated into an o/w nanoemulsion where the surfactants and cosurfactants not only help stabilization of nanoemulsion but also enhance the wettability of hydrophobic materials by getting deposited on the surface of the drug particles hence facilitating water penetration into the particle core [64]. Furthermore, the nanodroplet size of the nanoemulsion increases the total surface area where the size range of 10 to 100 nm is stated to be a dynamic microstructure enhancing the better drug solubilization and rapid diffusion into the aqueous medium [65]. The increased surface area results in a higher interfacial area required for the dissolution of hydrophobic drugs formulated into a nanoemulsion system [66, 67]. The *in vitro* drug release data was fitted into Weibull kinetic model, where the *b* value obtained was found to be less than 1 (*b* = 0.647, *r*^2^ = 0.9866, Figure 4(b)), which depicted that there was quick dissolution that resulted in the burst release of the drug from nanoemulsion system. Moreover, a steeper concentration gradient facilitated the process of drug diffusion and release [68].

### 5.6. Wound Contraction Analysis

**Figure 5:**
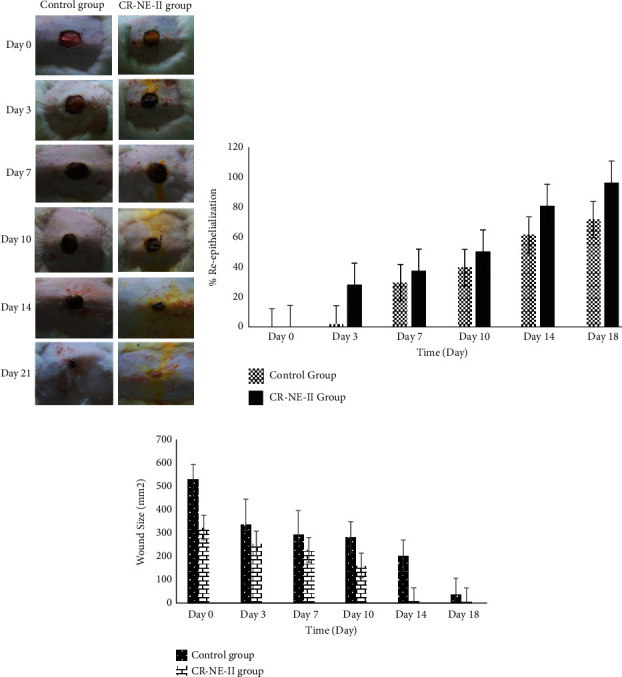
(a) Macroscopic wound images of diabetic rats with untreated and CR-NE-II-treated group. (b) Percent reepithelization. (c) Profile of wound size reduction.

The photographs of the wound healing period of the control and experimental (CR-NE-II) groups taken on days 0, 3, 7, 10, 14, and 21 are shown in Figure 5(a). The percent wound size significantly and fastly reduces and translated into significantly high percent reepithelization (Figure 5(b)) in the case of the CR-NE-II group in comparison to the control group (Student's *t*-test, *p* < 0.05). The percent reepithelization calculated on day 14 was found to be 96.47% in comparison to the control group (60.89%). The time required for complete wound closure and droppage of wound scab in the CR-NE-II group was 18 days, while the same was found to be 26 days in the control group. The CR-NE-II application onto the wound is envisaged to hasten the skin regeneration by exerting local antioxidant, antimicrobial, and hypoglycemic effects.

These results were also reported in previous studies. A curcumin-loaded self-emulsifying drug delivery system was fabricated for treating wound in diabetic animals. The in vivo results depicted that the curcumin-loaded SEDDS group had significantly early wound closure with higher degree of reepithelialization compared with the control and pure curcumin-treated groups [69]. In another study, curcumin nanoparticles and *α*-lactalbumin were used in combination for the treatment of diabetic wound infected with resistant microbial species like methicillin-resistant Staphylococcus aureus MRSA which significantly proved better topical treatment option for chronic wounds [70]. Furthermore, Bulbake et al. evaluated wound healing properties of curcumin incorporated composite graft of gelatin/PLGA microparticles using diabetic wound model. *In vivo* wound healing studies revealed that composite graft (Gel-Cur-cPMS) shows rapid wound contraction as compared to the Gel-Cur and control groups [71]. On the other hand, the *α*-tocopherol is also involved in the wound healing process by remodeling and prevention of hypertrophic scarring [35] and through activation of the expression of CTGF [72]. Thus, curcumin and vitamin E are envisaged to hasten the skin regeneration process by exerting synergistic antioxidant and anti-inflammatory effects locally in diabetic animal models.

### 5.7. In Vivo Microbial Contamination Test

To assess the microbial contamination propensity during the course of the wound healing process, sterile cotton swabs were gently rubbed into the wound and cultured on presterilized nutrient agar plates on day 3, day 7, day 14, and day 21 to assess the antimicrobial effect of CR-NE-II in comparison to the control group (supplementary Figure S1). The control group exhibited a higher number of microbial colonies formation than the CR-NE-II group. The CR-NE-II group also prevented significant microbial infiltration until days 3 and 7, while no microbial growth was seen in the wounds of diabetic animals that received nanoemulsion application. The increased antibacterial activity of CR-NE-II nanoemulsion can be attributed to the better interaction with the bacterial cell wall resulting in increased contact time and enhanced drug transport inside the bacteria [73]. Diabetic wounds are often complicated by secondary bacterial infection [66]. Therefore, the wound healing plate form with the ability to promote skin tissue regeneration but not allowing opportunistic bacteria to infiltrate and grow in the wound bed is detrimental to hastening wound healing in diabetics.

### 5.8. Skin Histology

**Figure 6:**
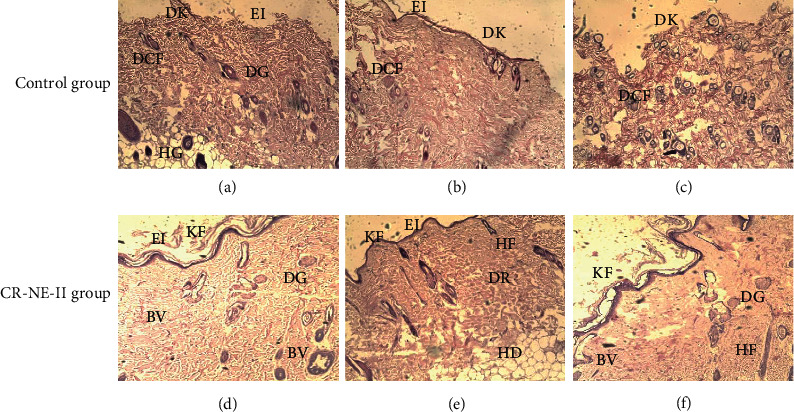
Hematoxylin and eosin staining 14-day diabetic wounded tissues of control group and CR-NE-II-treated group. KF: keratin formation; DK: dekeratinization; EI: epidermal integrity; DR: dermis; HD: hypodermis; DCF: degeneration of connective tissue fibers; DG: dermal gland; BV: blood vessels; HF: hair follicles.

Hematoxylin and eosin staining was performed on day 14 on skin samples excised from various animal groups (Figure 6) to evaluate the structural integrity of the stratum corneum (neoepidermis) inflammatory cell infiltration, blood vessel proliferation, and tissue granulation formation [74]. The control diabetic group sample showed slow migration of epithelial cells over the dermis layer due to a high concentration of inflammatory cells infiltration leading to dekeratinization (Figure 6(a)).

The CR-NE-II animal group showed complete reepithelialization due to rapid migration of epithelial cells from the basement membrane to uppermost layer and hence facilitated the healing process by faster wound closure and rapid scar formation. The epidermis was fully grown, and stratified squamous epithelium was shown in different layers of squamous cells, round cells at the bottom, and more flattened cells at the base of the epithelium (Figures 6(d) and 6(d)). The connective tissue fibers were properly arranged with many newly grown connective tissues, as evident in entire dermal tissue in fiber bundles. The dermal glands were fully grown and surrounded by different connective tissue fibers. The attachment of these fibers with the attached base of the dermal gland showed good healing characteristics, as the new fibers formed in the tissue showed proper healing properties (Figure 6(f)). A significant difference was observed in the histological architecture of all skin layers in the nanoemulsion group and control group. The curcumin nanoemulsion formulated with vitamin E exerted significant anti-inflammatory activity in diabetic wound application due to its proangiogenic nature [75]. These progressive changes appeared in the skin epidermal and dermal architecture include keratinization formation and migration of epithelial cells to regenerate stratum corneum, followed by higher reepithelialization. The early and increased migration of fibroblasts and inflammatory cells towards the site of injury in diabetic animals translates into suppression of inflammatory mediators, resulting in the fast generation of lost matrix and its deposition, and reduction in the inflammatory response in later stages helps tissue regain rapid maturation [76].

**Figure 7:**
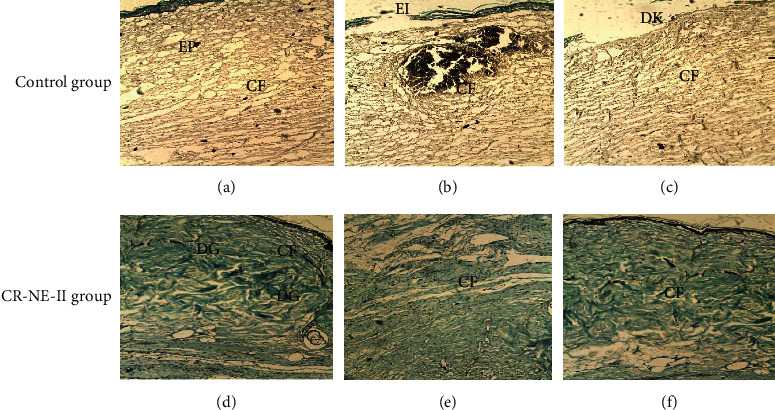
Masson trichome staining of 14-day wounded rat skin of control group and CR-NE-II group. EP: epidermis; SE: subepidermis; DR: dermis; EI: epidermal integrity; DK: dekeratinization; CF: collagen fibers; DG: dermal glands; BV: blood vessels.

The collagen fiber deposition results are shown in Figure 7. The histological analysis revealed that the CR-NE-II group (Figures 7(d)–7(f)) resulted in an abundant number of newly formed collagen fibers evenly distributed throughout the skin structure, especially around the dermal gland region and surrounding the hair follicles. Faster and even collagen matrix formation and deposition with smooth, elastic, well-aligned, and dense collagen fibers were observed in the CR-NE-II group, while the control group exhibited poor collagen deposition (Figures 7(a)–7(c)). Curcumin has been reported to enhance collagen matrix deposition by enhancing fibroblast proliferation and modulating the granulation tissue formation, which facilitates the production of extracellular matrix at the wound site [77]. Similarly, moisture retention on the wound site facilitates rapid regeneration of skin tissue [78]. This was why the CR-NE-II nanoemulsion application resulted in the formation of more evenly distributed collagen fibers throughout the skin microstructure compared to the control animal group.

### 5.9. Skin Vibrational Spectroscopic Analysis

**Figure 8:**
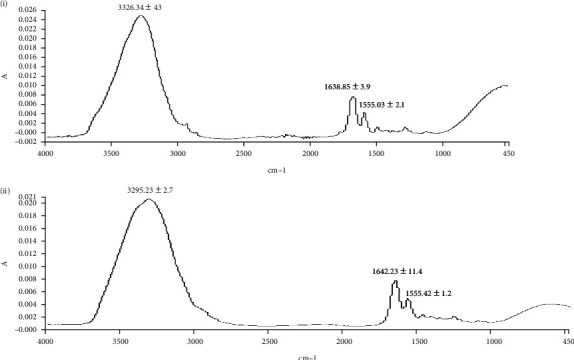
ATR-FTIR spectra of dermal layer of the (a) control group and (b) CR-NE-II group.

The skin samples excised on day 14 were also subjected to vibrational spectroscopic analysis to elucidate the charges incurred in the skin microstructure through protein propensity during the healing process elicited by various treatments. The results are shown in Figure 8. For the purpose, the dermis layer of the skin was analyzed on ATR-FTIR, and various corresponding bands were recorded with emphasis on absorbance for OH/NH (3326 to 3295 cm^−1^) and amide-I (1642 to 1638 cm^−1^) and amide-II (1555.03 to 1555.42 cm^−1^) which are signature bands originating from peptide linkages of the collagen [79]. The absorbance ratios of these regions in using control skin samples as a standard were used as a novel approach to elucidate protein deposition in the skin sample-treated group. The absorbance ratio of the CR-NE-II-treated group to the control group was found to be 0.96 ± 0.01 for OH/NH region while 1.46 ± 0.4 for amide-I and amide-II, which were significantly high (Student's *t*-test, *p* < 0.05) in comparison to the control group.

Spectral bands in vibrational spectra are molecule specific and provide direct information about the biochemical composition. FTIR peaks are relatively narrow and, in many cases, can be associated with the vibration of a particular chemical bond (or a single functional group) in the molecule [80]. In ATR-FTIR, absorbance is related to the concentration of a particular chemical component as stated by Beer-Lambert Law [81]. Therefore, higher absorbance ratio of a particular functional group indicates the higher intensity of that specific region. In this study, the fingerprint region shows a higher absorbance ratio of bands appearing at 1642 cm^−1^ (amide-1) and 1555 cm^−1^ (amide-II) in CR-NE-II compared to the control group, which indicates that more collagen protein is deposited at the wound site of curcumin nanoemulsion [82].

### 5.10. Tensile Strength

The skin samples extracted on day 14 of the control wound healing period and CR-NE-II animal group were subjected to stress break analysis, and the results are described in Table 5. The CR-NE-II application resulted in a significant increase in the mechanical strength of the skin containing wound on day 14 (Student's *t*-test, *p* < 0.05) in comparison to the control group. The strength of skin samples is dependent on the quantity of collagen matrix, being the major structural protein in skin, deposited as a function of time [83]. The uronic acid and hexosamine are the major matrix molecules which provide a necessary ground substrate for the new extracellular matrix, which are found at higher levels in the initial stages of wound healing while dropping down to normal in subsequent stages [84]. These components translate into the high activity of fibroblasts involved in the generation of substrate for the collagen fibers to be laid down [85]. Curcumin has been reported to increase uronic acid and hexosamine levels until the 8^th^ day of the wound healing period [86]. The higher levels of uronic acid at the wound site attract more fibroblasts and thus stimulate collagen synthesis by providing more fluid, which ensures higher cell mobility and early remodeling and helps hasten skin regeneration [84]. Similar results was reported by Basit et al. who developed composite film dressing of sodium alginate and pectin crosslinked with microwave and chitosan-curcumin nanoparticles for burn wound healing purposes. The nanoparticle-film combined application hence translated into a significant rise in the tensile strength as well as percent elongation break in comparison to untreated as well as other experimental animal groups, which is envisaged to be due to synergistic action of curcumin and nanoparticles in ensuring rapid formation of collagen fibers and their uniform deposition at wound site [87]. In the instant case, the animals treated with curcumin nanoemulsion showed higher mechanical strength on day 14, where a 2- to 2.5-fold increase in the tensile strength of the skin samples was observed compared to the control animal group.

### 5.11. Skin Thermal Analysis

**Figure 9:**
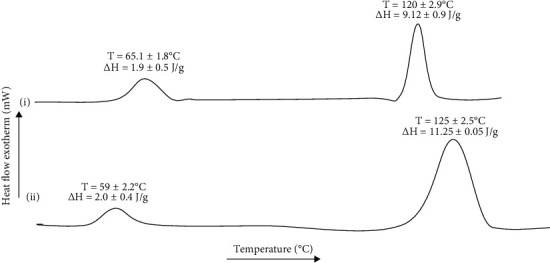
DSC thermogram of diabetic rat skin: (a) control group and (b) CR-NE-II group.

The thermal analysis is a widely employed analytical technique for estimation of changes in the melting temperatures and energy required to induce phase transition of lipidic and proteinous domains in the skin [88]. The thermal analysis results of day 14 skin samples of the control and CR-NE-II-treated groups are shown in Figure 9. The lipidic domains of the skin samples showed melting temperature in the range of 59-65°C with corresponding enthalpies in the range of 1.9 to 2.0 J/g. No significant difference (Student's *t*-test, *p* > 0.05) was observed among the control and CR-NE-II-treated animal groups in their thermal behaviour of lipidic domains, which reflects that skin lipid regeneration was independent of treatment applied [89]. In contrast, the proteinous domains of the skin samples of the CR-NE-II group underwent significant changes (Student's *t*-test, *p* < 0.05) in the melting temperature and the enthalpies of the system compared to the control group. The CR-NE-II-treated group sample showed a significant increase in the melting temperature of up to 125 ± 2.5°C in comparison to the control group of 120 ± 2.9°C. Curcumin has been reported to hasten skin protein deposition following injury [90]. The increase in transition temperature, as well as enthalpies of the curcumin-treated animal group, reflects more uniform collagen fiber deposition with increased crosslinking [24], which is also evident in a significant increase in the enthalpies of the curcumin-treated animal group samples where a significant increase of up to 11.25 J/g was observed (Student's *t*-test, *p* < 0.05). Enthalpy represents the energy required to induce phase transition [91]. The increased transition temperature and enthalpies with curcumin nanoemulsion treatment are hence envisaged to hasten collagen fiber synthesis and their uniform deposition following injury in diabetic animal models.

## 6. Conclusion

In this project, a novel nanoemulsion-encapsulating curcumin was formulated and evaluated for its wound healing potential in diabetic animals. The optimized formulation (CR-NE-II) showed size in nanorange and was homogenously dispersed with enough surface charge to ensure stability. The formulation showed a burst drug release pattern, which remained constant throughout the experiment. The novel nanoemulsion system exerted synergistic antioxidant and antibacterial activity. The optimized formulation hastened the skin tissue regeneration with significantly higher percent reepithelization and rapid wound closure in comparison to the untreated animal group, where an increased extent of collagen fiber deposition and reduced inflammatory response with high mechanical strength were observed. The curcumin-*α*-tocopherol nanoemulsion is hence advocated to be the most suitable formulation for wound healing applications in diabetic animals.

## Figures and Tables

**Table 1:** Curcumin nanoemulsion formulations.

Formulations	Tween-80 (g/g)	PEG-400 (g/g)	Vitamin E (g/g)	Curcumin (g/g)	Water (g/g)
BN-NE	12 g	12 g	3 g	0	73 g
CR-NE-I	12 g	12 g	3 g	0.3 g	72.7 g
CR-NE-II	10 g	14 g	3 g	0.3 g	72.7 g
CR-NE-III	6 g	18 g	3 g	0.3 g	72.7 g

**Table 2:** Droplet size, surface charge, and polydispersibility index of CR-NE formulations.

Formulation	Average droplet size (nm)	Polydispersity index (PDI)	Surface charge (ɀ) (mv)
CR-NE-I	19.66 ± 0.3	0.407 ± 0.02	−0.034 ± 1.85
CR-NE-II	26.76 ± 0.9	0.456 ± 0.04	−10.86 ± 1.06
CR-NE-III	63.7 ± 1.9	0.508 ± 0.002	−0.831 ± 1.43

**Table 3:** Drug content of curcumin nanoemulsion (CR-NE) formulations.

Formulation	Drug content (%)	Weight percentage
CR-NE-I	62.85 ± 1.1	0.129
CR-NE-II	68.05 ± 1.2	0.204
CR-NE-III	65.42 ± 0.9	0.138

**Table 4:** MIC of nanoemulsion with and without curcumin against *Pseudomonas aeruginosa* and *Staphylococcus aureus.*

Formulation code	MIC (*μ*g/ml)	Microbial species
CR-NE-II	187.5	*Pseudomonas aeruginosa*
CR-NE-II	93	*Staphylococcus aureus*
BN-NE	Not active (at highest tested 1.5 mg/ml)	Not active against both species

CR-NE = curcumin nanoemulsion, BN-NE = blank nanoemulsion.

**Table 5:** Mechanical properties of rat skin samples.

Skin samples	Tensile strength (MPa)	Elongation break (%)	Elastic modulus (MPa)
Control group	5.428 ± 2.2	16.777	1.7143
CR-NE-II group	10.435 ± 2.1	14.714	3.2857

## Data Availability

The data could be excessed and made available by contacting the corresponding author (if required).

## Supplementary Materials

Supplementary Materials Figure S1: images of microbial growth at different days of wound healing in the untreated (control) and CR-NE-II-treated groups of diabetic rats.
