# Supplementary material for: Novel Curcumin-Encapsulated α-Tocopherol Nanoemulsion System and Its Potential Application for Wound Healing in Diabetic Animals
Source: Biomed Res Int. 2022 Sep 15;2022:7669255. doi: 10.1155/2022/7669255 (PMC9499807; doi:10.1155/2022/7669255)
Supplement: Supplementary Materials — Figure S1: images of microbial growth at different days of wound healing in the untreated (control) and CR-NE-II-treated groups of diabetic rats. [file 7669255.f1.docx]

**Supplementary file**


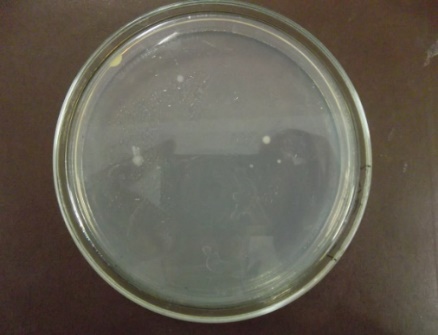

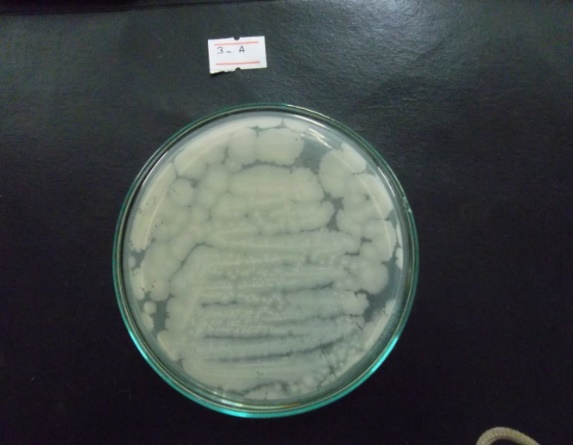

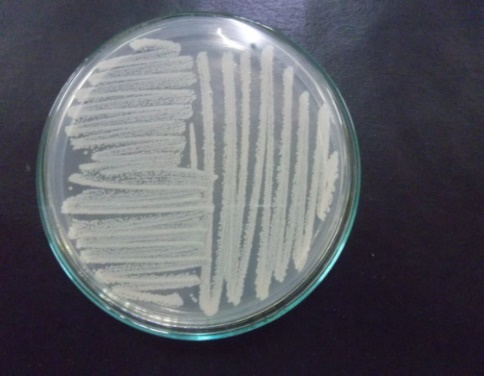

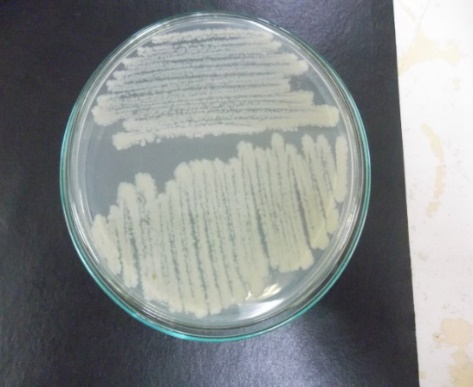

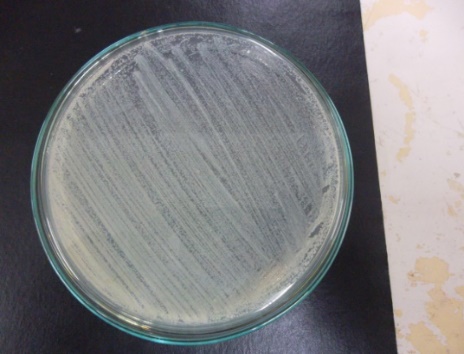


**Day 3**

**Day 7**

**Day 14**

**Day 21**


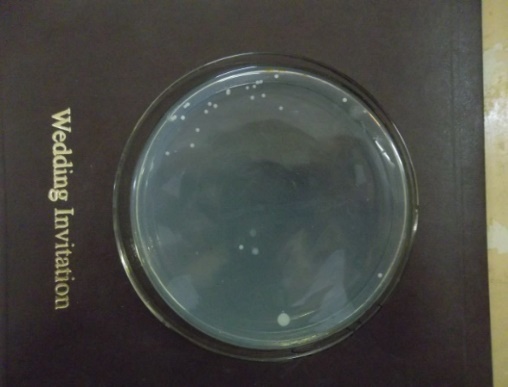

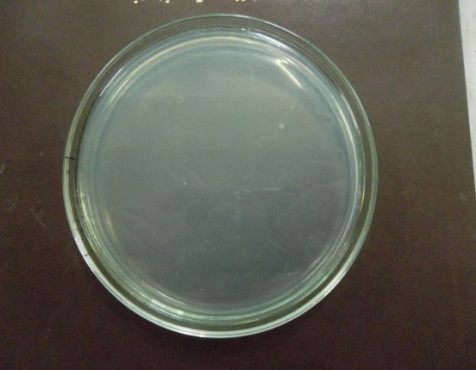

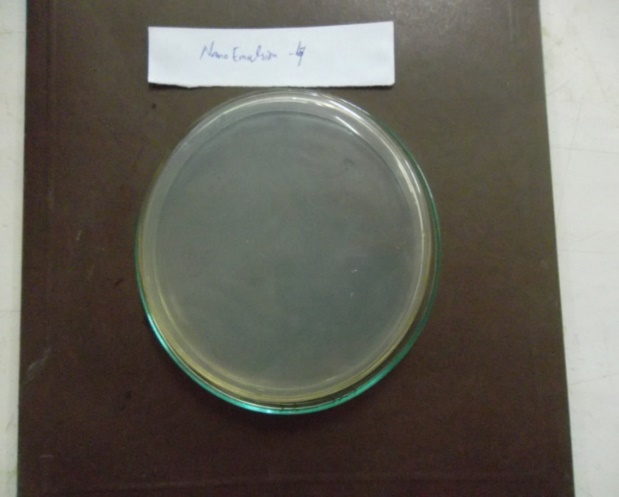


**Control Group**

**CR-NE-II Group**

FIGURE 1S: Images of microbial growth at different days of wound healing in untreated (control) and CR-NE-II treated group of diabetic rats
